# Partnering with Family Organizations in Research: Experiences from the Field

**DOI:** 10.3390/ijerph22060894

**Published:** 2025-06-03

**Authors:** Jessica Holmes, Tennyson Dahlman, Eric J. Bruns, Misty Woody, Melissa Hendricks, Millie Sweeney, Bruno J. Anthony

**Affiliations:** 1 Department of Psychiatry, University of Colorado School of Medicine, Mail Stop F550 1890 N. Revere Court, Anschutz Health and Sciences Building, Suite 5240, Aurora, CO 80045, USA; jessica.holmes@cuanschutz.edu (J.H.); tennyson.dahlman@cuanschutz.edu (T.D.); 2 Department of Psychiatry, University of Washington School of Medicine, Seattle, WA 98195, USA; ebruns@uw.edu; 3 Allegheny Family Network, 1501 Reedsdale Street Suite 2007, Pittsburgh, PA 15233, USA; mwoody@alleghenyfamilynetwork.org; 4 Oregon Family Support Network, 4275 Commercial St. SE, Suite 180, Salem, OR 97302, USA; melissah@ofsn.net; 5 Family-Run Executive Director Leadership Association, 4725 Dorsey Hall Dr a316, Ellicott City, MD 21042, USA; msweeney@fredla.org; 6 Pediatric Mental Health Institute, Children’s Hospital Colorado, Mail Stop A036/B130, 13123 E. 16th Ave., Aurora, CO 80045, USA

**Keywords:** Parent Peer Support, Family-Run Organizations, participatory action research, mental health, systems of care, Nominal Group Process

## Abstract

Parent Peer Support (PPS) provided by other caregivers who have lived experience raising a child with mental health conditions (e.g., such as anxiety, depression, attention, and/or behavior disorders) holds promise as a service that can improve outcomes by helping young people and their families overcome system- and individual-level barriers to receiving care. Here, we describe the development of a collaboration of researchers and Family-Run Organizations (FROs) to provide research support for PPS through three phases (1) developing a research agenda and study aims through a Patient-Centered Outcomes Research Institute (PCORI) “Pipeline to Proposal” grant; (2) designing a pilot study, including refining the measurement framework for a successful grant application to the National Institute of Mental Health (NIMH); and (3) implementing the study through the incorporation of research methodology into FRO operations without disrupting the organizations’ missions. This paper summarizes the participatory action research (PAR) strategies employed by this interdisciplinary research team throughout the three phases, covering the benefits and challenges of these unique partnership activities. We focus on how this project was able to increase the relevance of the research to the FROs and the communities they serve and improve dissemination and utilization of the results to support other PPS projects.

## 1. Introduction

Parent Peer Support (PPS) is an effective and advantageous service that can aid in improving both child/youth and caregiver outcomes by overcoming system- and individual-level barriers to receiving care [1,2]. Typically delivered as a supplement to other services such as individual and family therapy and/or care coordination, PPS is delivered by current or former parents or caregivers with lived experience raising children with identified mental health needs. Key elements of PPS include the provision of information, aid in navigating systems, emotional support, and direct advocacy [3,4]. The PPS theory of change posits that peer support partners aid in the reduction of caregiver strain by having an ally and provider of emotional support. Their credibility with parents also allows them to engender trust and thus assist parents in becoming more actively engaged in their child’s services, leading to greater follow-through and engagement in needed services [1,5,6].

Although currently available in some form in nearly every state in the U.S. [7], the level of research support for and types of outcomes associated with PPS is scant. Given the expansion of PPS nationally, its increased inclusion as a billable service in state Medicaid plans [8], and its promise as a method of supporting parent/caregiver wellness as well as a method to increase engagement in other services, additional research is crucial. Collecting data about and formally studying PPS also is critical to the continued growth and implementation of this valuable type of support.

Ideally, research on PPS will fully engage both those providing the support (trained caregivers) and those receiving the support (caregivers and their families). Engaging those receiving services and their families in healthcare research as participants is beneficial in many ways—increased study enrollment rates, designing study protocols, development of more effective interventions and responsive policies, and involving them as co-researchers that contribute to knowledge, help refine approaches, and build essential relationships for innovative change [9]. Such participatory action research (PAR) also aligns with broader historical movements toward more democratic modes of inquiry that involve partners at all levels [10,11]. In addition to improving the overall quality of research and engagement with participants, PAR is also proposed to increase the relevance of research among communities and improve dissemination and utilization of the research findings [12,13].

However, this engagement can be challenging for researchers because of logistics such as time and funding needed for engagement, accessing different types of caregiver knowledge, traditional power hierarchies, and concerns around tokenistic engagement [11,13,14]. An innovative approach to mitigating these challenges in mental health research efforts is to partner with Family-Run Organizations (FROs), non-profit organizations with governing boards of at least 50% caregivers with lived experience caring for a child or youth with mental health needs, and whose administration and staff are mainly caregivers with lived experience. FROs are natural conduits and trusted cultural brokers for families and in their communities. There are over 100 local and statewide FROs that, collectively, reach over 100,000 families annually through training, family support, and system navigation for children’s behavioral health issues [15]. With decades of experience providing PPS, FROs are currently the best source of technical assistance, consultation, training, and family-driven service provision across the United States [16]. They, too, benefit from participating as collaborators in research as the data can be used to inform practice, policy, and advocacy efforts in their states.

Here, we describe a PAR process specific to PPS. An interdisciplinary team of national, local, and state FRO leaders, local PPS providers, parents with lived experience, and mental health researchers embarked on a federally funded study of PPS providers (2019–2023) focusing on elementary-school aged children with emerging behavioral health concerns in two states. The study team included the Family-Run Executive Director Leadership Association (FREDLA), a grassroots network of Family-Run Organizations (FROs) across the country dedicated to supporting families caring for a child/youth with behavioral health needs, as well as two FROs in Pennsylvania (Allegheny Family Network) and Oregon (Oregon Family Support Network).

This paper summarizes the PAR strategies employed by this interdisciplinary research team across three phases of grant-funded efforts: (1) developing a research agenda and study aims, (2) designing a pilot study including refining measures specific to the study, and (3) implementing the research methodology integrated into FRO procedures. Successfully achieving these phases required managing details of the partnership, through collaborative efforts to address barriers to conducting the research within these unique organizations and in the context of the COVID-19 pandemic. Within each of these sections, we will provide examples of unique partnership activities, effective strategies, specific outputs, and recommendations that can be replicated in other sites and projects.

## 2. Phases of Collaboration

### 2.1. Developing a Research Agenda

#### 2.1.1. Convening a National PPS Research Partnership

In 2015, FREDLA applied for and was awarded a “Pipeline to Proposal” grant from PCORI. The resulting “Family Research Partnership” leveraged existing and new relationships among parent leaders, family members, researchers, and mental health providers to build a national advisory group focused on defining a research agenda for PPS. The Partnership was co-led by co-authors of this paper, including FREDLA’s Director of Learning and Workforce Development, a parent of children with emotional and behavioral challenges, and researchers specializing in youth mental health services with lived experience as a parent or foster parent of youth with mental health issues.

The governance structure for the Partnership defined expectations for shared leadership and decision-making based on community-based participatory research principles [11,12] and a commitment to transparent, respectful communication. A key principle was that the Partnership would adhere to the values and philosophy of Systems of Care [16], the foundation of which states that, “…parents/caregivers will be equal partners in all decisions that affect their children as well as policies and processes that affect all children”. Basing the Partnership on these values helped to establish a foundation of equality, shared decision-making, and respect for the lived experience of parents and caregivers. The governance document also dictated that all decisions would be made by consensus, respecting the input of all members and their areas of expertise. The Partnership was committed to honoring and respecting the intellectual property of all members, and it was agreed upon that information would be used only within the project to reach its objectives.

#### 2.1.2. Determining Research Priorities

The Partnership obtained additional funding from PCORI and expanded to include representatives from national organizations, managed care organizations, and members of the national PPS provider workforce nominated by local and state FROs. With this growth, the governance document was amended to state that Partnership composition would comprise no less than 50% family members and family leaders and that recruitment would be facilitated through the networks of FREDLA’s FROs and other partnership members’ connections to public youth- and family-serving systems. The primary goal for a PCORI “Pipeline to Proposal” award is to produce one or more high-quality research proposals. To achieve this goal, the Partnership used several methods to engage partners in a process of defining priorities and developing research questions. Starting with a core advisory group of family leaders and researchers, the group identified what was missing from the current literature on PPS. The group comprised four researchers (two of whom were also parents), three FREDLA staff members (who were also family leaders and PPS providers), and nine family leaders/PPS providers from across the nation for a total of 16 members. The Partnership also developed and disseminated surveys via several FROs to elicit families’ experiences with PPS. From this information, the co-leaders generated top PPS priorities and recommendations using the Nominal Group Technique (NGT) [17].

After the first several steps of the process, which involved individual brainstorming, round-robin sharing of ideas, and consolidation of ideas into themes, each member of this advisory group voted for the top three themes that resonated most with them. As shown in Table 1, group members prioritized the need for research on the effectiveness of receiving PPS services compared to receiving behavioral health services without PPS. The same 16 members mentioned in the previous paragraph voted on the themes shown in Table 1, Table 2 and Table 3.

The Partnership went on to conduct two more rounds of NGT (*n* = 16) to determine the most important outcomes (see Table 2) and the most important considerations (see Table 3).

In sum, the Partnership used broad-based stakeholder recruitment combined with techniques aligned with the need for consensus building to gain critical information in identifying research priorities and outcomes for PPS as well as set the tone for a truly participatory process. In response to the top priorities, outcomes, and considerations, the Partnership then submitted and received funding for an Exploratory Research Grant (R34) to the National Institute of Mental Health (NIMH) in 2019, titled *Navigation and Parent Peer Support to Promote Access and Retention of Children in Mental Health Services.* The primary aims of the study were to: (1) refine the FREDLA PPS model, which included the addition of measuring fidelity, quality, feasibility, and acceptability; and (2) design and conduct a small-scale randomized trial evaluating outcomes of behavioral health services for youth with emotional behavioral concerns (EBC) and their caregivers when paired with PPS providers using the refined PPS Practice Model versus no PPS. These developments and the implementation of that project are described in the following sections.

### 2.2. Collaboratively Designing a Pilot Study

#### 2.2.1. Identifying Partners

A respectful community-based research project actively engages partners in ways that ensure their voices and perspectives are valued and integrated while prioritizing transparency, mutual respect, and equitable power dynamics. It was important for community partners to recognize the benefits of collaborating in research, such as designing research questions and useful tools to obtain critical data for use in advocacy. This collaborative model, characterizing the present research, involved responsibilities and challenges for the community FRO partners in identifying research questions and developing methodology, including measurement structures, data collection, and interpretation. It also required participation in decision-making throughout the project. 

Aware of these expectations, two FRO members of the Family Research Partnership, the Allegheny Family Network (AFN) and UPLIFT Wyoming, volunteered to participate in the research project and were key partners in refining the goals of the research and how those goals would be achieved. However, shortly after the project began, Uplift Wyoming, had to leave the project because they lost funding for one of the two PPS providers involved in the project and was replaced by the Oregon Family Support Network (OFSN), a statewide organization. Discussions were held with OFSN leadership to ensure that they agreed with the focus and importance of the project goals and the requirements of participating organizations. The aim was to make clear what research partners aimed to achieve and the strategies that needed to be developed to align those goals in ways that ensure mutually beneficial research outcomes.

#### 2.2.2. Refining the PPS Model and Quality Indicators

Research partner FROs engaged in review and feedback on the package of PPS training and implementation support, utilizing the phases of PPS established in the FREDLA Practice Model. The model, based on decades of PPS provision by FROs, existing research, and best practices from the field, provides competency-based training in PPS focusing on necessary skill sets for each phase of PPS (see Table 4). The model also includes training for supervisors in using a developmental approach to supervision and the nuances of supporting a lived experience workforce. Trainers and licensed sites receive coaching on the provision of the curriculum and ongoing support of both PPS providers and their supervisors.

Grant development also focused on confirming and/or developing relevant measures. The research team had proposed a set of measures related to the study aims, which were then merged with tools related to key FRO goals. Indeed, it is often the case that the data that interests researchers may not directly interest or apply to FROs, requiring give and take on both sides. For the FROs, a key task was to develop a rigorous measure of adherence to the PPS model to allow the providers to make adjustments to the service and inform training. While there are several tools used by those providing PPS to measure or track outcomes (e.g., caregiver engagement in services, caregiver strain, youth symptoms), no tool exists specific to measuring the quality and/or adherence of PPS. To address this need, the team developed and began testing the Parent Peer Support Quality Indicator (PPS-QI). To help with this process, the research team had to be open to learning from the PPS providers about the role they play with families. The tool was designed to be used by FROs to support PPS providers in supervision, coaching, and continuous quality improvement, as well as with parents/caregivers after PPS services to measure the quality of support received. The item pool for the PPS-QI was drawn from FREDLA’s Practice Model and was reviewed by the Parent Advisory Committee of the Allegheny Family Network and seasoned parent support partners (e.g., PPS providers) who were trainers in the PPS Practice Model.

To further assess the impact of PPS, the project incorporated the Family Journey Assessment (FJA) [5] which was developed by PPS providers who collaborated with researchers to assess PPS providers’ success in facilitating parents’ reduction of the need for help on specific skills and care plan implementation. The FJA, completed by PPS providers and family members together, tracks caregiver progress toward self-advocacy and self-efficacy with a three-component structure: (1) reflecting progress in the recognition of needs, (2) collaboration to access help from formal and natural supports, and (3) activation of skills to cope with stress, enhance resilience, and develop and carry out plans of care. FROs eagerly worked to embed this tool into their database forms and fields which was ideal for tracking FJA data. The FROs hosted two trainings for the FJA with ongoing refresher trainings throughout the project for new staff.

#### 2.2.3. Developing the Research Design

The initial goal of the design was to evaluate the impact of PPS by randomly assigning youth/caregivers to PPS provided by staff with lived experiences raising a child with mental health conditions and who were trained in the FREDLA Practice Model or to receive only resources from a local Family Support Organization that provided information and referrals by staff who were not family members with lived experience or trained PPS providers. Initially, the plan was to work with local school personnel who would provide a mechanism for self-referral at school functions and referrals from school staff. A school liaison was to collect referral information and serve as a bridge with the research team. However, a few weeks after the onset of project recruitment, the COVID-19 pandemic hit and had a profound impact on the local school districts. Schools were closed to mitigate the spread of the virus and then switched to virtual and hybrid learning depending on the infection rates. Schools were confronted with unprecedented challenges in providing equitable access for all students, supporting teachers’ and students’ educational needs, and making plans amidst great uncertainty. As a result, participating schools were less able to focus on referring children to the study. Also, with virtual learning, parents and teachers interact less and teachers have less direct contact with students, making it more difficult to identify those children with developing mental health issues, which was the focus of our study.

A new recruitment method was developed, employing our FRO research partners’ help lines—AFN’s Parent Support Line and OFSN’s Reach Out Oregon. Both services receive calls from parents referred through a variety of pathways (e.g., schools, community organizations, social media) and are staffed by trained individuals who provide information, find connections to community services, help negotiate systems, and give emotional support. For both FRO help lines, families were given the choice to receive support from an FRO PPS provider trained in the FREDLA Practice Model, rather than being randomly assigned to the resource-only condition, where they would not have received support from a trained PPS provider. This change in design was supported by the partner FROs, fitting more clearly with their mission. Because PPS providers are trained to offer support, enhance caregiver skills, and empower families, randomly assigning a caregiver to the resource-only condition went against their core values which meant that families would not receive PPS services if they asked for them. As a result, the research team pivoted to a quasi-experimental design to examine the impact of the extent of PPS participation on service use and youth/caregiver outcomes.

## 3. Integrating Research Methodology

### 3.1. Developing Research Procedures

The research team and both FROs worked together to create a reasonable workflow that incorporated project procedures without disrupting the organization’s mission, particularly the work of the PPS providers. To do that, both FRO partners (AFN and OFSN) provided a clear understanding of the PPS work to allow the most seamless integration and to minimize the extra workload on staff. For example, AFN carefully presented the project to staff, planned step-by-step what needed to be added or changed, and who would be involved, and what their roles would be. An internal team from both AFN and OFSN was created, and they met monthly with the research team to discuss obstacles and challenges. These monthly meetings were also a time to ask questions and share successes. Other integration strategies included a shared email and file-sharing group, monthly “All Hands In” meetings, biweekly data team meetings, a staff newsletter, and marketing templates for articles, press releases, and flyers. Additionally, all research documentation and materials were approved through the FRO Parent Advisory Board, a group of caregivers trained to support the mission and work of AFN and provide feedback on all aspects of AFN’s structure and services. This helped in keeping the documents parent-friendly, attractive, and understandable as participating in research projects can be intimidating and daunting to the parents we serve. We also asked staff to review materials and documentation to ensure we were not complicating or contradicting their current processes.

A key process that needs to be developed collaboratively is participant recruitment, including how participants learn about the study and decide whether they would like to participate, without disrupting internal processes. In our work, procedures needed to be carefully specified as to how participants were put in contact with the research team and how it might impact interactions or rapport between the participants and FRO staff. Gaining input from FRO staff through joint meetings with researchers was critical to the successful development of eligibility criteria and parent recruitment procedures.

### 3.2. Setting Boundaries and Delegating Roles

Community partners must understand the commitment involved in research studies. For our work, fully discussing and agreeing upon FRO responsibility as the collaboration was formed, and continuing to do so throughout, helped clarify expectations and set boundaries in roles. Every project will vary in FRO responsibilities, ranging from being a simple referral resource to being a full partner in the project. While the research team establishes certain project parameters based on the funding and IRB guidelines, the FRO team is on the ground and, therefore, often serves as the key partner tasked with troubleshooting challenges in real-time.

To help set boundaries and define roles, the FROs appointed a point of contact. This staff member communicated the project’s purpose, its alignment with the FRO mission and core values, and facilitated regular internal meetings. They also met regularly with the research team and were informed of any changes before implementation. The point of contact was also instrumental in serving as a champion for our research project. Research teams partnering with FROs and PPS providers need to understand that it takes time to develop a rapport with your research partners. They are extremely passionate about the support they provide families. Because they have lived experiences with their own children like those of the caregivers to whom they’re providing PPS to, they are empathetic, protective, and deeply care about the well-being of the families. We experienced pushback, resistance, and skepticism from PPS providers at first. This was completely understandable considering we were asking them to do quite a lot, especially not offering PPS to caregivers who were randomly assigned to the resource-only condition. The point of contact was able to meet with PPS providers and explain to them this research could benefit their organization directly and their role as a PPS provider. It can’t be stressed enough how essential it was to have a champion within the FROs. They were a trusted staff member, and their enthusiasm and commitment to the project eventually spilled over to the PPS providers.

### 3.3. Mutual Understanding

Researchers need to be open to learning from their community partners about the work they do. In our project, it was important to understand the PPS provider’s role and the dynamic with families to validate and respect the FRO perspective. For example, it was the case in our work that the research project required PPS providers to do work on top of their full-time jobs. Remaining patient and flexible with the FRO and PPS providers was key to success. When starting a research project, there needs to be clear and constant communication between the research team and the FRO staff involved in the project. First, goals should be clearly defined for what both partners aim to achieve, and strategies should be developed to align those goals in ways that ensure mutually beneficial research outcomes. Second, essential information on the implementation of the project needs to be communicated. This includes where research activities will occur, how the research team will collect the information and data necessary to assess outcomes, who the participants in the research will be, and what type of data will be collected. From this starting point, the communication established early in the study will be a necessary component throughout its course and essential to the success of the research.

In our project, we were mindful that families who were seeking support from FROs were likely experiencing challenges and/or crises. Additionally, there still exists a stigma associated with mental health conditions and seeking treatment, whether it’s for oneself or their family [18,19]. Recruitment and retention in mental health trials can be challenging. Time, patience, and persistence are necessary when recruiting families who are experiencing crises. Experience has shown that it can be effective to reach out to families multiple times, send frequent reminders about measures to fill out, provide multiple means of getting in touch with the researchers (e.g., phone and email), and be prepared to consent and help with measures outside of normal work hours.

At times, families won’t be the only participants in this type of research. Researchers are interested in FRO processes and learning more about the interactions the PPS providers have with families. This PPS perspective can also help make sense of the results of studies with families. It is essential to include PPS providers’ feedback in research, which is why, at times, they’ll also be asked to be participants themselves.

### 3.4. Establishing and Maintaining Communication

Clear avenues of communication need to be developed and maintained given that processes may need to change as the research continues. The research team needs to become very familiar with the organization and the people involved and be prepared to explain and re-explain the purpose and benefits of the research to any employee involved in the study. We found that setting up weekly or biweekly meetings with PPS providers and FRO team leaders helped facilitate relationship building and trust between the researchers and employees at the FRO. These meetings helped keep both the research team and FROs engaged, allowed the FROs to ask questions, and prompted conversations about changes in processes. Meetings were also a great time to check in to see which things were working and going well. In addition to meetings, principal investigators should also consider visiting the FRO site throughout the project to engage and connect with the PPS providers and leadership helping to run the study.

We found that identifying a knowledgeable liaison at the FRO ensured that we were not making impossible demands. The liaison advocated for necessary changes and asked for clarification if there was confusion surrounding any research process or activity. Of note, the PACS team distributed a monthly newsletter with updates about enrollment, any changes that had been made, and notes of encouragement.

### 3.5. Continued Collaboration

Research partnerships with community organizations like FROs continue well past the data collection phase. In our case, we closely collaborated with FROs to analyze and interpret the data, especially since the FROs provided our research team access to data that they internally track in their databases and records. Because they shared their data with us, it was vital that the FROs continued to assist in translating the outcomes, identifying how and where to disseminate, and partnering for conference presentations or scholarly articles. The research team and FROs collaboratively developed a plan to present findings to the FROs’ staff members to inform current service delivery, adopt and implement the PPS Practice Model, and develop evidence-based programs and trainings. The results may provide support for FRO funding proposals and grant applications to strengthen and expand their services. The FRO team added important context to the findings so they would not be misinterpreted. FROs and PPS providers intimately know their families, their work, and their data best. This is why they needed to ensure the results were relevant and determine the impact on other organizations.

## 4. Discussion

PPS provided by other caregivers who have lived experience raising a youth with MH conditions, holds promise as a service that can help improve outcomes by helping young people and their families overcome system- and individual-level barriers to care. The number of professional PPS providers serving parents and caregivers of children with mental health needs is rapidly increasing and over half of all states in the United States now provide Medicaid reimbursement for this service [20]. Although there has been progress toward greater specification of PPS program models and core competencies, there remains scant research on the effective implementation of such strategies in real-world settings. Here we described the development of a health services research study of the impact of PPS implemented in two states and employing a PAR framework to further the support for PPS services. Success rested on several significant strategies.

First, key learning from our work with FROs was the importance of helping these community partners recognize the benefits of collaborating in research, such as designing research questions, goals, and useful tools to obtain critical data for use in advocacy. We found it was important to be upfront about how the data would be used and shared, addressing important concerns and a priority for our partners. In particular, FROs were eager to evaluate the reach and implementation of the FREDLA version of PPS, which aims to improve rates of service access, engagement and retention, and a range of caregiver and youth outcomes. Also, AFN strongly supported the development of the quality indicators in order to make adjustments to the service and service model and training.

Multiple factors may complicate the evaluation of community-based efforts, particularly in situations where the randomized controlled trial (RCT) is culturally unacceptable, resulting in the use of a non-randomized trial. This was the case in our work where all caregivers were offered PPS but not all accepted or carried through with the service. As a result, analyses had to carefully consider potential biases utilizing statistical methods to control for confounding variables. With this design, we made sure that our partners understood the limitations and possible biases and that the results may not be as conclusive as a randomized controlled trial (RCT). Also, key aspects of the analytic strategies included the identification of potential confounding variables that could influence the outcome and methods of controlling them through statistical adjustments or matching. We decided to rely on propensity score methods to evaluate the probability of treatment level given confounders [21,22]. It has been shown that creating balance in the distribution of the propensity score between treatment groups will on average balance the variables that went into the estimation of the propensity score model. Therefore, the use of propensity score methods can emulate how randomization balances baseline factors in an RCT. However, it is important to note that propensity score methods balance only those factors measured and included in the propensity score model, while randomization theoretically balances all factors, both measured and unmeasured.

We learned the importance of open and transparent dialogue between researchers and community members, recognizing unique expertise and perspectives throughout all stages of the research project. Specifically, we provided participating families with multiple means of communication to maintain involvement in the research study. With our FRO partners, we held regular meetings focusing on project progress and actively shared information, concerns, and perspectives to build trust, ensure equitable participation, and collaboratively guide the research direction to address community needs effectively. Listening to one another was viewed as essential for strong collaboration in other studies to understand both partners’ perspectives and foster trust in the research and the partnership.

Building quality, long-term research relationships comes down to trust. Adaptations and changes to the processes of a project are inevitable in community-based research. Researchers should understand that method and design plans cannot be rigid, but rather, should be viewed as dynamic. We found it critically important to listen, seek consent, and incorporate feedback from the FRO leadership and staff in key decisions. They often have the best insight into what is working and what is not.

## 5. Conclusions

This paper describes a community partnership research approach, involving the joint expertise of organizational representatives, community members, and researchers that led to the successful development and implementation of a project to evaluate the impact of a growing part of the mental health care continuum—Parent Peer Support. The project demonstrated the importance of creating a formal partnership and gathering information through a group process from all partners to determine research priorities and key outcomes. The success of the collaborations required community partners to understand the benefits of the research project. In this case, FROs worked to finalize the research goals, questions, and useful tools (e.g., fidelity and quality measures) to obtain critical data to enhance the effectiveness of their work. For the FROs, a key task was to develop a rigorous measure of adherence to the PPS model to allow the providers to adjust the service and inform training. At the same time, it was important for the research team to understand and appreciate the goals, functions, and services of the FROs for successful collaboration. Indeed, the FROs strong belief that all caregivers and youth should receive PPS services if asked for, led to the use of a quasi-experimental design to examine the impact of the extent of PPS participation. The team also worked hard to incorporate project procedures without disrupting the organization’s mission. Biweekly meetings with research and FRO team leaders to exchange information, resolve differences, implement needed changes, and build trust and engagement led to important shared decision-making, ensuring that the research addresses their needs and leads to meaningful, sustainable change. This approach fosters trust, builds capacity, and leads to more effective solutions. Most importantly, these joint efforts led to funding opportunities to further support the development and understanding of this effective and valued support for families.

## Figures and Tables

**Table 1:** Results of Group Process Identifying Priorities for PPS Research (*n* = 16).

Most Important Priorities	Number of Votes	Percentage of Votes
What is the effectiveness of behavioral health services for families with versus without receipt of PPS	16	100%
Does PPS initiated earlier in the course of the youth’s behavioral health problem promote better outcomes than PPS initiated later?	10	63%
Within the primary care context, does PPS promote more positive youth and family outcomes?	9	56%
How does the modality of delivery (e.g., remote/telephonic versus in-person) affect the effectiveness of PPS?	8	50%
How does the lived experience (amount, type) of PPS provider affect outcomes?	6	38%

**Table 2:** Results of Group Process Identifying Outcomes for PPS Research (*n =* 16).

Most Important Outcomes	Number of Votes	Percentage of Votes
Social determinants—home security, food security, chronic health, parenting in a safe manner	13	81%
Family empowerment/self-efficacy/sense of competence	11	69%
Parental stress/strain/depression	10	63%
Reduced need for more intensive/residential/inpatient services/ER visits	9	56%
Collaboration with services/service use and increase in natural supports	9	56%

**Table 3:** Results of Group Process Identifying Additional Considerations for PPS Research (*n =* 16).

Most Important Additional Considerations	Number of Votes	Percentage of Votes
Ensure PPS model is well-defined and specifies PPS is delivered by those with lived experience and embedded in a Family-Run Organization	14	88%
Choose a comparator carefully to make sure Comparative Effectiveness Research facilitates PPS status as an evidence-based practice	12	75%
Focus on families who have experienced trauma	9	56%
Match PPS provider to family demographics and clinical characteristics	6	38%
Be sure research design can enable examination of outcome for different times of initiating PPS (e.g., at start vs. mid-stream in services)	5	31%

**Table 4:** FREDLA Skill Sets for PPS.

PPS Phase	Focus and Associated Skill Sets
Connect	Presenting self as a peer and establishing a role with family
Discover	Understanding the family level of needs, strengths, and goals
Support	Support of family across systems, including developing and implementing a support plan with tasks and building collaborative relationships
Empower	Empowering families and informing systems around family perspective, family voice and choice, and family-driven services
Prepare	Transitioning from formal support, including the development of the ongoing plan for support and acknowledging skills learned
Take Care	PPS Provider self-care and maintaining role

## Data Availability

Data from the NIMH R34 grant (entitled *Navigation and Parent Peer Support to Promote Access and Retention of Children in Mental Health Services*, R34MH119431) can be obtained with permission through the NIMH Data Archive (Clinical Trial ID NCT04029220).

## Abbreviations

The following abbreviations are used in this manuscript:

PPS	Parent Peer Support (PPS)
FRO	Family-Run Organization
PCORI	Patient-Centered Outcomes Research Institute
NIMH	National Institute of Mental Health
PAR	Participatory Action Research
NGT	Nominal Group Technique
FREDLA	Family-Run Executive Director Leadership Association
PPS-QI	Parent Peer Support Quality Indicator
FJA	Family Journey Assessment
AFN	Allegheny Family Network
OFSN	Oregon Family Support Network
